# Characterization of *BRCA1/2* mutations in patients with family history of breast cancer in Armenia

**DOI:** 10.12688/f1000research.10434.1

**Published:** 2017-01-10

**Authors:** Sofi Atshemyan, Andranik Chavushyan, Nerses Berberian, Arthur Sahakyan, Roksana Zakharyan, Arsen Arakelyan

**Affiliations:** 1 Institute of Molecular Biology, Armenian National Academy of Sciences, Yerevan, Armenia; 2 ARTMED Medical Rehabilitation Center (CJSC), Yerevan, Armenia

**Keywords:** breast cancer, BRCA1, BRCA2, mutation screening, targeted exome sequencing

## Abstract

**Background. **Breast cancer is one of the most common cancers in women worldwide. The germline mutations of the *BRCA1* and *BRCA2* genes are the most significant and well characterized genetic risk factors for hereditary breast cancer. Intensive research in the last decades has demonstrated that the incidence of mutations varies widely among different populations. In this study we attempted to perform a pilot study for identification and characterization of mutations in *BRCA1* and *BRCA2* genes among Armenian patients with family history of breast cancer and their healthy relatives. **Methods. **We performed targeted exome sequencing for *BRCA1* and *BRCA2* genes in 6 patients and their healthy relatives. After alignment of short reads to the reference genome, germline single nucleotide variation and indel discovery was performed using GATK software. Functional implications of identified variants were assessed using ENSEMBL Variant Effect Predictor tool. **Results. **In total, 39 single nucleotide variations and 4 indels were identified, from which 15 SNPs and 3 indels were novel. No known pathogenic mutations were identified, but 2 SNPs causing missense amino acid mutations had significantly increased frequencies in the study group compared to the 1000 Genome populations. **Conclusions. **Our results demonstrate the importance of screening of *BRCA1* and *BRCA2* gene variants in the Armenian population in order to identity specifics of mutation spectrum and frequencies and enable accurate risk assessment of hereditary breast cancers.

## Introduction

Breast cancer (BC) is one of the most common cancers in females worldwide ^1^ and particularly in Armenia ^2^. Despite the high prevalence of this disease in developed countries, it has become highly prevalent in developing countries (50% of all cancer cases) and is characterized by high mortality rate (58% of all breast cancer related deaths) ^3^.

The germline mutations of the *BRCA1* ^4^ and *BRCA2* ^5^ genes are the most significant and well characterized genetic risk factors for hereditary breast cancer, which constitutes about 5–10% of all cases ^6^. Inherited mutations in *BRCA1* and *BRCA2* genes account for 30–50% of all known mutations associated with this disease ^7, 8^. Women who carry *BRCA1* mutations are particularly susceptible to the development of breast cancer before the age of 35–40 with a probability rate of 45%–60%, whereas women who inherit a *BRCA2* mutation have a 25%–40% risk of developing breast cancer ^7, 8^. The association of *BRCA1/BRCA2* gene mutations with breast cancer was first well described in Ashkenazi Jews ^8– 11^. Intensive research in the last decades has demonstrated that the incidence of mutations in high-risk families varies widely among different populations ^6^. For example, the mutations in *BRCA1* and *BRCA2* were each estimated to account for 45–50% of families with multiple cases of breast and ovarian cancer in UK and USA ^3, 12^, whereas mutation prevalence among African–Americans with family breast and ovarian cancer history was 16.3% for *BRCA1* and 11.3–14.4% for *BRCA2* ^13, 14^, which is significantly lower compared to Caucasian populations. Identification of the *BRCA1/BRCA2* mutations in different populations and ethnic groups is an important endeavor, which enables geneticists and oncologists to make more specific choices in genetic testing of members of high-risk families ^15– 17^.

Here we have attempted to perform a pilot study for identification and characterization of mutations in *BRCA1* and *BRCA2* genes among Armenian patients with family history of breast cancer and their healthy relatives.

## Materials and methods

### Samples

Six patients with confirmed family history of breast cancer (at least two cases in a family) and their first-degree healthy relatives were recruited in this study (except for the BC10 patient, see Table 1). Patients were admitted to the National Center of Oncology MH RA and ARTMED Medical Rehabilitation CJSC. Written informed consent forms were obtained from all the study participants. This study was approved by the Institutional Review Board (IRB00004079) of the Institute of Molecular Biology NAS RA.

**Table 1.** Family structure of the studied subjects.

Family	Sample	Agec (Age at Diagnosis)	Disease	Family history	Healthy relative in study
FAM1	BC01	67	BC	Yes (daughter)	BC02
FAM1	BC02		HC		
FAM2	BC03	41	BC	Yes (sister [BC04], grandmother)	BC05
FAM2	BC04	40	BC	Yes (sister [BC03], grandmother)	BC05
FAM2	BC05		HC		
FAM3	BC06	42	BC	Yes (mother, aunt)	BC07
FAM3	BC07		HC		
FAM4	BC08	36	BC	Yes (aunt)	BC09
FAM4	BC09		HC		
FAM5	BC10	38	BC	Yes (mother, aunt)	NA

Blood samples were collected in EDTA-containing tubes and genomic DNA was extracted according to the protocol described elsewhere ^18^. A260/A280 ratio measured for evaluation of quality and quantity of extracted DNA was in the range of 1.8–2.

### Exome sequencing

*BRCA1* and *BRCA2* exome sequencing was performed by an external service provider (Admera Health LLC, South Plainfield, NJ, USA) using the proprietary breast cancer panel iBRCA ^TM^, which detects genetic variations in all exons of *BRCA1* and *BRCA2*. According to the service provider’s description, this panel utilizes the targeted amplicon (166 amplicons) sequencing method, based on Seq-Ready™ TE Panels protocol (WaferGen Biosystems Inc, Freemont, CA, USA). Reagent cocktails and samples were aliquoted into a 384-well sample source plate. The source plate and BRCA1/2 SmartChip™ were pre-dispensed with Seq-Ready™ TE BRCA1/2 Primers and were placed into the SmartChip™ Multisample Nanodispenser. The SmartChip™ was then amplified with Bio-Rad T100 SmartChip™ TE Cycler. PCR product was then purified with Agencourt AMPure XP (Beckman Coulter, Inc.), according to manufacturer’s instructions. Samples were then quantified with Qubit® 2.0 Fluorometer (Thermo Fisher Scientific, Inc.) and quality analyzed with Tapestation (Agilent Technologies). Sequencing was performed with Illumina MySeq platform on a single lane. Raw reads for each sequenced sample were stored in separate fastq files. DNA samples were shipped on ice to avoid degradation and were passed internal quality check before processing.

### Short-read alignment

For each sample, raw sequences were aligned to the human reference genome sequence (hg19, see *Public genome data section*) using Burrows-Wheeler Aligner (BWA) version 0.7.10 with default parameters. The resulting bam files were used in downstream variant discovery analysis.

### Variant discovery

Variant discovery was performed using Genome Analysis Tool Kit (GATK) version 3.6 according to recommended workflows for germline single nucleotide variations (SNVs) and indel discovery in whole genome and exome sequencing data ^19^. Base quality score recalibration, indel realignment and mate pair fixing were performed in bam files. Variant calling was performed without duplicate read removal. SNV and indel discovery and genotyping were performed simultaneously across all samples using standard hard filtering parameters ^19^.

### Public genome data

For the alignment, we have used the human reference genome sequence (NCBI build 36.1/hg19) from the UCSC (University of California, Santa Cruz) database ( http://genome.ucsc.edu). Known SNPs (single nucleotide polymorphisms) were annotated using the UCSC database (single nucleotide polymorphism database, dbSNP version 135). 1000 Genomes phase 1 genotype data was used for human genetic variations filtration ( ftp://ftp.1000genomes.ebi.ac.uk/vol1/ftp/). Allelic frequencies of detected variants were compared against 1000 Genomes phase 3 genotypes, as well as with the genome-wide association study (GWAS) data from 54 healthy Armenian females that were genotyped in the framework of population genetics study by Harber *et al.* ^20^ ( ftp://ngs.sanger.ac.uk/scratch/project/team19/Armenian). The Data on clinically significant *BRCA1* and *BRCA2* variants were obtained from Breast Cancer Core DataBase maintained by National Human Genome Research Institute ( https://research.nhgri.nih.gov/bic/).

### Statistical analysis and functional annotation

Comparison of allele frequency distributions in the study group with 1000 Genomes and healthy Armenians was performed using Fisher’s exact test available in R 3.3.2 base package. Variant functional annotation was performed using ENSEMBL Variant Effect Predictor tool ^21^.

## Results

In this study we have performed exome sequencing of *BRCA1* and *BRCA2* genes in patients with a positive family history of breast cancer and their healthy relatives of Armenian origin. Patients’ clinical data and family structure of the studied subjects are presented in the Table 1. The aligned sequencing data is available in the NCBI Sequence Read Archive (SRA, https://www.ncbi.nlm.nih.gov/sra/) under accession SRP095082. For each sample, a total of 166 different primer pairs were used to amplify all the coding regions of *BRCA1* and *BRCA2* (as described in the Methods section). The average sequencing depth per base per sample was 6696±606. Detailed NGS statistics are presented in Table 2 and Supplementary file S1.

**Table 2.** Overall NGS statistics.

Number of samples	10
Total aligned reads (percent aligned reads)	1106492 (90%)
Target aligned reads (percent aligned reads)	1041136 (94%)
Mean coverage depth	6696.9
Target coverage at 1×	100%
Target coverage at 10×	99.99%
Target coverage at 50×	99.95%

In total, variant calling resulted in detection of 232 sequence variations (200 SNVs and 32 indels, Supplementary datasets S2 and S3). Thirty-nine SNVs and 4 indels passed the thresholds after applying hard filters ( Table 3).

**Table 3.** Polymorphic variants in *BRCA1* and *BRCA2* genes in patients and their healthy relatives. This table provides functional annotation of mutations in *BRCA1* and *BRCA2* genes that passed filters during variant calling with GATK.

HGVSg	Consequence	Impact	Gene	HGVSp	SIFT	PolyPhen	Clinical significance
13:g.32889775 G>T	5' UTR	MD	*BRCA2*	-	-	-	-
13:g.32889792 A>G	5' UTR	MD	*BRCA2*	-	-	-	-
13:g.32890572 G>A	5' UTR	MD	*BRCA2*	-	-	-	-
13:g.32899159 C>T	intronic	MD	*BRCA2*	-	-	-	uncertain significance, not provided
13:g.32900933 T>A	intronic	MD	*BRCA2*	-	-	-	-
13:g.32906729 A>C	missense	MO	*BRCA2*	p.Asn372His	tolerated	benign	not provided, benign
13:g.32910561 A>G	missense	MO	*BRCA2*	p.Glu690Gly	deleterious	possibly damaging	-
13:g.32910594 T>G	missense	MO	*BRCA2*	p.Phe701Cys	tolerated	benign	-
13:g.32911888 A>G	synonymous	L	*BRCA2*	p.Lys1132Lys	-	-	benign
13:g.32913055 A>G	synonymous	L	*BRCA2*	p.Leu1521Leu	-	-	benign, likely benign
13:g.32913081 A>G	missense	MO	*BRCA2*	p.Lys1530Arg	deleterious	probably damaging	not provided
13:g.32913609 A>G	missense	MO	*BRCA2*	p.Asn1706Ser	tolerated	benign	uncertain significance
13:g.32914236 C>T	missense	MO	*BRCA2*	p.Thr1915Met	tolerated	benign	benign
13:g.32918825 T>C	intronic	MD	*BRCA2*	-	-	-	-
13:g.32920905 T>C	intronic	MD	*BRCA2*	-	-	-	-
13:g.32929232 A>G	synonymous	L	*BRCA2*	p.Ser2414Ser	-	-	benign
13:g.32929387 T>C	missense	MO	*BRCA2*	p.Val2466Ala	tolerated	benign	uncertain significance, benign
13:g.32929451 A>G	intronic	MD	*BRCA2*	-	-	-	-
13:g.32936646 T>C	intronic	MD	*BRCA2*	-	-	-	benign
13:g.32953388 T>C	intronic	MD	*BRCA2*	-	-	-	not provided
13:g.32973012 A>C	3' UTR	MD	*BRCA2*	-	-	-	uncertain_significance
17:g.41216021 G>A	synonymous	MD	*BRCA1*	-	-	-	uncertain significance
17:g.41223094 T>C	missense	MO	*BRCA1*	p.Ser1634Gly	tolerated	benign	not provided, benign, likely benign
17:g.41226601 G>C	intronic	MD	*BRCA1*	-	-	-	-
17:g.41231516 C>T	intronic	MD	*BRCA1*	-	-	-	-
17:g.41234470 A>G	synonymous	L	*BRCA1*	p.Ser1436Ser	-	-	-
17:g.41244000 T>C	missense	MO	*BRCA1*	p.Lys1183Arg	tolerated	benign	benign
17:g.41244429 C>T	missense	MO	*BRCA1*	p.Ser1040Asn	tolerated	benign	benign
17:g.41244434 T>C	synonymous	L	*BRCA1*	p.Glu1038Glu	-	-	-
17:g.41244644 T>A	synonymous	L	*BRCA1*	p.Pro968Pro	-	-	-
17:g.41244936 G>A	missense	MO	*BRCA1*	p.Pro871Leu	tolerated	benign	not provided
17:g.41245237 A>G	synonymous	L	*BRCA1*	p.Leu771Leu	-	-	benign
17:g.41245466 G>A	synonymous	L	*BRCA1*	p.Ser694Ser	-	-	benign
17:g.41245471 C>T	missense	MO	*BRCA1*	p.Asp693Asn	deleterious	benign	benign
17:g.41249324 A>G	intronic	MD	*BRCA1*	-	-	-	uncertain significance, likely benign
17:g.41251906 T>A	intronic	MD	*BRCA1*	-	-	-	-
17:g.41251931 G>A	intronic	MD	*BRCA1*	-	-	-	not provided
17:g.41256032 G>C	intronic	MD	*BRCA1*	-	-	-	-
17:g.41256037 C>T	intronic	MD	*BRCA1*	-	-	-	-
13:g.32913172 delC	frameshift	H	*BRCA2*	-	-	-	-
17:g.41249364 delA	intronic	MD	*BRCA1*	-	-	-	-
17:g.41256076 delA	intronic	MD	*BRCA1*	-	-	-	-
17:g.41256087_41256101 delGAAAAAAAAAAGAAA	intronic	MD	*BRCA1*	-	-	-	-

HGVSg – genomic position of mutation notation by Human Genome Variation Society; Consequence – consequence of mutation; Impact – functional impact of mutation (MD – modifier, MO – moderate, L – low, H – high); HGVSp - protein sequence name notation by Human Genome Variation Society; SIFT - prediction of protein function change depending on amino acid substitution using SIFT software ( http://sift.jcvi.org/); PolyPhen - prediction of protein function change depending on amino acid substitution using PolyPhen software ( genetics.bwh.harvard.edu/pph2/).

From these variants, 18 were novel (15 SNV and 3 indels), and the rest have already been described in 1000 Genomes populations ( Table 4). The novel variants were detected only in one or two subjects (8 in healthy relatives and 7 in patients). We identified 12 missense variants (5 in *BRCA1* and 7 in *BRCA2*), 8 synonymous variants (5 in *BRCA1* and 3 in *BRCA2*), 15 intronic variants (8 in *BRCA1* and 7 in *BRCA2*) and 4 in untranslated regions of *BRCA2*. The frequency distributions of known *BRCA1/2* variants were similar to those in 1000 Genomes populations and/or GWAS of healthy Armenians, except for the g.32914236 C>T (p _Fisher_=8.35E-24 vs Armenians, p _Fisher_=0.013 vs 1000 Genomes) and g.41245471 C>T (p _Fisher_=0.013 vs Armenians, p _Fisher_=4.7-E05). No known clinically significant variants were detected in breast cancer patients and their healthy relatives.

**Table 4.** Distribution of identified variants in healthy Armenians and in 1000 genomes populations. The frequency distributions of identified mutations in the study group were compared with data from 1000 Genomes population, as well as the genome-wide association study from 54 healthy Armenian females ^20^.

HGVSg	RAF	MAF	RAF 1000 Genomes	MAF 1000 Genomes	RAF Armenians	MAF Armenians
13:g.32889775 G>T	0,90	0,10	-	-	-	-
13:g.32889792 A>G	0,75	0,25	0,85	0,15	-	-
13:g.32890572 G>A	0,75	0,25	0,79	0,21	0,73	0,27
13:g.32899159 C>T	0,95	0,05	-	-	-	-
13:g.32900933 T>A	0,70	0,30	0,65	0,35	-	-
13:g.32906729 A>C	0,70	0,30	0,75	0,25	0,72	0,28
13:g.32910561 A>G	0,95	0,05	-	-	-	-
13:g.32910594 T>G	0,95	0,05	-	-	-	-
13:g.32911888 A>G	0,70	0,30	0,73	0,27	0,71	0,29
13:g.32913055 A>G	0	1	0,03	0,97	-	-
13:g.32913081 A>G	0,95	0,05	-	-	-	-
13:g.32913609 A>G	0,95	0,05	-	-	-	-
13:g.32914236 C>T	0,90	0,10	0,99	0,01	0,98	0,02
13:g.32918825 T>C	0,95	0,05	-	-	-	-
13:g.32920905 T>C	0,95	0,05	-	-	-	-
13:g.32929232 A>G	0,70	0,30	0,77	0,23	-	-
13:g.32929387 T>C	0	1	0,02	0,98	0	1
13:g.32929451 A>G	0,95	0,05	-	-	-	-
13:g.32936646 T>C	0,60	0,40	0,47	0,53	0,64	0,36
13:g.32953388 T>C	0,60	0,40	0,49	0,51	0,63	0,37
13:g.32973012 A>C	0,60	0,40	0,84	0,16	0,70	0,30
17:g.41216021 G>A	0,95	0,05	0,99	0,01	-	-
17:g.41223094 T>C	0,50	0,50	0,64	0,36	-	-
17:g.41226601 G>C	0,50	0,50	0,65	0,35	-	-
17:g.41231516 C>T	0,50	0,50	0,65	0,35	-	-
17:g.41234470 A>G	0,50	0,50	0,66	0,34	0,55	0,45
17:g.41244000 T>C	0,50	0,50	0,65	0,35	0,55	0,45
17:g.41244429 C>T	0,95	0,05	0,99	0,01	0,98	0,02
17:g.41244434 T>C	0,90	0,10	-	-	-	-
17:g.41244644 T>A	0,95	0,05	-	-	-	-
17:g.41244936 G>A	0,50	0,50	0,46	0,54	0,53	0,47
17:g.41245237 A>G	0,50	0,50	0,66	0,34	0,55	0,45
17:g.41245466 G>A	0,50	0,50	0,66	0,34	0,61	0,39
17:g.41245471 C>T	0,70	0,30	0,97	0,03	0,92	0,08
17:g.41249324 A>G	0,95	0,05	-	-	-	-
17:g.41251906 T>A	0,95	0,05	-	-	-	-
17:g.41251931 G>A	0,80	0,20	0,90	0,10	0,78	0,22
17:g.41256032 G>C	0,95	0,05	-	-	-	-
17:g.41256037 C>T	0,95	0,05	-	-	-	-
13:g.32913172 delC	0,95	0,05	-	-	-	-
17:g.41249364 delA	0,50	0,50	0,67	0,33	-	-
17:g.41256076 delA	0,25	0,50	-	-	-	-
17:g.41256087_41256101 delGAAAAAAAAAAGAAA	0,50	0,18	-	-	-	-

MAF – minor allele frequency; RAF – reference allele frequency.

## Discussion

This study provides preliminary characterization of variations in *BRCA1* and *BRCA2* genes in Armenian patients with family history of breast cancer. Our data suggest that no known clinically significant variants ^22^ contribute to the disease development in these patients. Meanwhile, two other frequent mutations were identified that cause missense substitutions in coding regions of *BRCA1* and *BRCA2* and were predicted as having pathogenic consequence. The results of this study are in agreement with a a previous report, which also failed to identify known high risk mutations of *BRCA1* and *BRCA2* genes in Armenian patients using high-resolution melting PCR approach ^23, 24^.

Mutations in *BRCA1* and *BRCA2* genes are known markers for hereditary breast/ovarian cancer ^25^. Currently more than 100 clinically important mutations and polymorphisms have been described. Genetic testing of these mutations was among the first included in the guidelines for cancer prognostics ^3, 4^. Nowadays, in many countries genetic testing is routinely prescribed to patients in high-risk groups for hereditary breast and ovarian cancer ^26– 28^. However, it has also become apparent that the distribution and appearance of particular risk alleles in *BRCA1* and *BRCA2* genes is population dependent, and in many cases population specific mutations are being identified ^8– 11^. This is especially relevant to populations that have for a long time remained culturally and genetically isolated ^8– 11^, as in the case of Armenians. Recent research has demonstrated that the genetic structure of Armenians “stabilized” about 4000 years ago and has remained almost unchanged since that time ^20^. Furthermore, our own data indicate that the frequencies of genetic variations associated with various complex human diseases share similarities both with European and Asian populations ^29– 31^. From the other side, Armenian genomes are highly underrepresented in the current human genome sequencing initiatives and little is known about genetic predisposition to complex diseases in this particular population.

In conclusion, despite the small sample size limitation, our results demonstrate the importance of screening of *BRCA1* and *BRCA2* gene variants in the Armenian population in order to identity specifics of mutation spectra and frequencies and enable accurate assessment of the risk of hereditary breast cancers.

## Data availability

The data referenced by this article are under copyright with the following copyright statement: Copyright: © 2017 Atshemyan S et al.

The aligned sequencing data is available in the NCBI Sequence Read Archive (SRA) under accession number SRP095082 ( https://www.ncbi.nlm.nih.gov/sra/?term=SRP095082). Scripts and vcf files with called and filtered genotypes are available: DOI, 10.5281/zenodo.215615 ^32^.

## Supplementary materials

**Supplementary File 1. Sequencing statistics: Coverage and target enrichment statistics.**

This file contains details on sequencing coverage and enrichment, which were extracted from the QC report compiled by Admera Health LLC, South Plainfield, NJ, USA.
