# Supplementary material for: Characterization of BRCA1/2 mutations in patients with family history of breast cancer in Armenia
Source: F1000Res. 2017 Jan 10;6:29. [Version 1] doi: 10.12688/f1000research.10434.1 (PMC5357036; doi:10.12688/f1000research.10434.1)
Supplement: Peer review history

# Peer review: Referee response for version 1

**DOI:** 10.5256/f1000research.11244.r21044

**Published:** 2017-03-17

**Authors:** Lusine Nazaryan-Petersen

**Affiliations:** 1 Department of Cellular and Molecular Medicine (ICMM), Faculty of Health Science, University of Copenhagen, Copenhagen, Denmark

This is a very important pilot study characterizing variations in BRCA1 and BRCA2 genes in Armenian patients with family history of breast cancer. It provides a good background for further large-scale study in Armenia.

 I have few notes to consider:

1. In the section Methods, the authors used 1000 Genomes phase 1 genotype data for variations filtration. Is there any reason why they prefer phase1 data but not phase 3, which they used for assessing allelic frequencies?
2. I noticed that the authors did not verify the NGS detected variants by other methods, e.g. by Sanger sequencing. It is especially important to confirm the detected novel mutations to exclude that they could be false positive.
3. In the Table 3, the authors report a frameshift variant 13:g.32913172delC, which has a high functional impact on *BRCA2*. Is it detected in a patient or in a healthy relative? Could it be a novel mutation specific for the Armenian population?  It is known that PolyPhen and SIFT may fail to predict the impact for some variants. The authors might consider to verify this mutation by other methods, e.g. Sanger sequencing, and report it to the appropriate databases. I would suggest to mention about this variant under the section Discussion.

I have read this submission. I believe that I have an appropriate level of expertise to confirm that it is of an acceptable scientific standard.

# Peer review: Referee response for version 1

**DOI:** 10.5256/f1000research.11244.r20365

**Published:** 2017-03-01

**Authors:** David A. Goukassian

**Affiliations:** 1 PhD Center for Translational Medicine, Temple University School of Medicine , Philadelphia, PA, USA

The breast cancer is an important health problem in Armenia and identifying specific genetic factors that may predispose to breast cancer development, especially in the families of patient that were already diagnosed with this condition may improve significantly the dire situation with breast cancer prevention in Armenia. Although in the small number of patients and family members, the manuscript presents a good step forward and sets an example how genetic studies in the larger cohort of breast cancer patients and members of their families could identify clinically relevant variants in BRCA1/2 gene mutations that known elsewhere outside of Armenian population as well identify variants that could be specific for Armenian populations only.

 The title of manuscript is appropriate and the abstract summarizes well the reported findings. Study design is appropriate, albeit with small number of patients. Materials and methods and data analyses are suitable for the design and conclusion are justified. Methodology provides sufficient information and references for replication of the experiments as well as to build up the data base with the larger cohort of patients and their family members.

 A few suggestions to make the discussion of the results better:

1. In this study there were no known clinically relevant variants identified. Could this be because of small number of patients in addition to the conceived notion that genetic structure of Armenians is “stabilized” 4000 years ago? Could there be other predisposing factors, as well? Needs a bit more discussion.
2. What is the value of the novel variants identified in this study? Could these novel variants be specific for Armenian population? Are there any other “close ethnic groups” that have shown novel variants that are not clinically relevant for the "mainstream population" but became relevant for the specific ethnic group. Brief discussion will suffice.

I have read this submission. I believe that I have an appropriate level of expertise to confirm that it is of an acceptable scientific standard.
